# Serological Diagnosis of Paracoccidioidomycosis: High Rate of Inter-laboratorial Variability among Medical Mycology Reference Centers

**DOI:** 10.1371/journal.pntd.0003174

**Published:** 2014-09-11

**Authors:** Monica Scarpelli Martinelli Vidal, Gilda Maria Barbaro Del Negro, Adriana Pardini Vicentini, Teresinha Inez Estivalet Svidzinski, Maria Jose Mendes-Giannini, Ana Marisa Fusco Almeida, Roberto Martinez, Zoilo Pires de Camargo, Carlos Pelleschi Taborda, Gil Benard

**Affiliations:** 1 Medical Mycology Laboratory (IMTSP and LIM-53), Clinics Hospital of the Medical School and Tropical Medicine Institute, University of São Paulo (USP), São Paulo, São Paulo State, Brazil; 2 Mycoses Immunodiagnosis Laboratory, Adolfo Lutz Institute, São Paulo (IAL-SP), São Paulo, São Paulo State, Brazil; 3 Medical Mycology Laboratory, Laboratory of Teaching and Research in Clinical Analysis from Maringá(LEPAC), Maringá, Parana State, Brazil; 4 Clinical Mycology Laboratory, Pharmaceutical Sciences School, São Paulo State University (UNESP), Araraquara, São Paulo State, Brazil; 5 Serology Laboratory, Clinics Hospital, Ribeirão Preto School of Medicine (FMRPUSP), São Paulo State, Brazil; 6 Myco-serology Laboratory, Department of Microbiology, Immunology and Parasitology, Federal University of São Paulo (UNIFESP), São Paulo, São Paulo State, Brazil; 7 Biomedical Sciences Institutes, Department of Microbiology, USP, São Paulo, São Paulo State, Brazil; University of California, San Diego, School of Medicine, United States of America

## Abstract

**Background:**

Serological tests have long been established as rapid, simple and inexpensive tools for the diagnosis and follow-up of PCM. However, different protocols and antigen preparations are used and the few attempts to standardize the routine serological methods have not succeeded.

**Methodology/Principal findings:**

We compared the performance of six Brazilian reference centers for serological diagnosis of PCM. Each center provided 30 sera of PCM patients, with positive high, intermediate and low titers, which were defined as the “reference” titers. Each center then applied its own antigen preparation and serological routine test, either semiquantitative double immunodifusion or counterimmmunoelectrophoresis, in the 150 sera from the other five centers blindly as regard to the “reference” titers. Titers were transformed into scores: 0 (negative), 1 (healing titers), 2 (active disease, low titers) and 3 (active disease, high titers) according to each center's criteria. Major discordances were considered between scores indicating active disease and scores indicating negative or healing titers; such discordance when associated with proper clinical and other laboratorial data, may correspond to different approaches to the patient's treatment. Surprisingly, all centers exhibited a high rate of “major” discordances with a mean of 31 (20%) discordant scores. Alternatively, when the scores given by one center to their own sera were compared with the scores given to their sera by the remaining five other centers, a high rate of major discordances was also found, with a mean number of 14.8 sera in 30 presenting a discordance with at least one other center. The data also suggest that centers that used CIE and pool of isolates for antigen preparation performed better.

**Conclusion:**

There are inconsistencies among the laboratories that are strong enough to result in conflicting information regarding the patients' treatment. Renewed efforts should be promoted to improve standardization of the serological diagnosis of PCM.

## Author Summary

Paracoccidioidomycosis (PCM) is a neglected systemic fungal infection prevalent mostly in South America. Serological tests have long been established as rapid, simple and inexpensive tools for the diagnosis and follow-up of PCM. However, different protocols and reagents are used. We compared here the performance of six Brazilian reference centers for serological diagnosis of PCM. Each center provided 30 sera of PCM patients, with positive high, intermediate and low titers, which were defined as the “reference” titers. Each center then applied its serological routine test to the 150 sera from the other five centers blindly as regards to the “reference” titers. Surprisingly, all centers exhibited a high rate of discordances (mean of 31 discordant scores in 150 sera tested). When the scores given by one center to their own sera were compared with the scores given to their sera by the other centers, a high rate of major discordances was found (a mean of 14.8 sera in 30 presented a discordance with at least one other center). We concluded that there are inconsistencies among the laboratories that can potentially result in conflicting information regarding the patient's treatment. Renewed efforts should be promoted to improve standardization of the serological diagnosis of PCM.

## Introduction

Paracoccidioidomycosis (PCM) is a neglected systemic fungal infection prevalent mostly in South America. Despite the significant progress in several areas of knowledge since it was described by Adolpho Lutz, in 1908, it still shows high rates of morbidity and mortality and low visibility [1]. In rural areas of Brazil there are approximately four new cases per million inhabitants, making it the third cause of death from chronic infections, with 1.65 cases per million [2].

The gold standard of PCM diagnosis is the visualization of yeast cells with typical multiple budding aspect in a clinical sample or isolation of the fungus in culture medium [3]. The latter has either low sensitivity when samples obtained from non-sterile sites (e.g., sputum) are used, or is more sensitive in sterile, deep-seated site samples, which, however, are not frequently available. In addition, the growth of *P. brasiliensis* can take several weeks [3], [4]. Serological tests have been established since the 70's contributing to the rapid, simple and inexpensive diagnosis of the mycosis [5]–[8].

Several antigenic preparations, including sonicated extracts and filtered phase concentrated of cultures of the yeast form of the fungus, have so far been used for the serological diagnosis of PCM [9]. Early on some authors reported on the issue of variability in the antigen preparations [10], [11]. The growth of yeast cells is performed in culture media and conditions such as incubation time, temperature, size of inoculum, with or without agitation, can lead to differences in the antigens produced in different diagnostic centers.

In fact, different protocols and antigen preparations are currently used by these centers for the serological diagnosis and follow up of patients with PCM. Most centers use semiquantitative immunoprecipitation techniques, either the double immunodiffusion (DID) or counterimmunoelectrophoresis (CIE), or both [7], [12]–[14]. However, their performance is not routinely checked, in part due to the lack of external standards. Only an internal positive control is used, which in most centers is a patient's serum with a known positive titer. Moreover, the few attempts put forward to standardize the routine serological methods used in PCM patients have not succeeded [15]. One major reason is that the reference centers have been carrying out in house methodologies for many years with apparently satisfactory performances [11], [15], [16]. However, unfortunately in most instances these centers do not have feedback regarding the clinical correlation from the physicians assisting the patients.

To address this issue, we compared the performance of laboratories from six medical mycology reference centers in Brazil that carry out routine serological diagnosis of PCM. The results show that there are inconsistencies among the laboratories, strong enough to result in conflicting information regarding the patient's treatment, and that renewed efforts should be promoted to improve standardization of the serological diagnosis of PCM.

## Materials and Methods

### Design of the study

Six reference centers that traditionally and routinely perform serological diagnosis of PCM participated in this study. They all have made significant scientific contributions to the improvement of the serological diagnosis of this mycosis and for that reason were invited to participate in the study: Mycoses Immunodiagnosis Laboratory, Adolfo Lutz Institute, São Paulo (IALSP); Myco-serology Laboratory, Department of Microbiology, Immunology and Parasitology, Federal University of São Paulo (UNIFESP); Clinical Mycology Laboratory, Pharmaceutical Sciences School, São Paulo State University (UNESP), Araraquara, SP; Serology Laboratory, Clinics Hospital, Ribeirão Preto School of Medicine of the University of São Paulo (FMRPUSP); Medical Mycology Laboratory, Laboratory of Teaching and Research in Clinical Analysis from Maringá State (LEPAC); and Medical Mycology Laboratory Clinics Hospital of the Medical School (LIM53) and Tropical Medicine Institute, University of São Paulo (IMTSP).

Each center was requested to provide 30 sera of PCM patients from their repository, with positive high, intermediate and low titers according to their own criteria. The anonymized sera were numbered 1–30 and aliquots of 120 µl were sent to the remaining five centers to perform their own serological assays. Thus each center performed their usual serological assays in 150 sera from 5 different centers blindly with regard to the “reference” titer of the sera. The results were then sent directly to the coordinating center (IMTSP), which analyzed the data. In addition, the coordinating center also provided all centers with aliquots of 6 healthy donor sera, as negative controls. These donors did not have previous history of tuberculosis or any other significant infectious disease, and the sera were non-reactive for PCM and histoplasmosis.

### Ethical statement

The study was approved by the Human Research Analysis Ethics Committee of the Hospital das Clínicas da Faculdade de Medicina da USP, accession number #7915.

### Serological assays

All centers employed an immunoprecipitating technique, either semiquantitative DID [17] or CIE [18]. The isolates used for antigen preparation are shown in Table 1. Two antigens were used: (a) the somatic antigen, obtained through sonication (100–150 W for 30′) of the cells grown for 15 days in Fava Netto's medium at 35°C [19] and (b) the culture filtrate (metabolic antigen), obtained from yeast cells grown in Negroni's medium for 7–10 days (log phase growth) at 37°C [11]. The sonicated antigen is kept frozen (−20°C) while the culture filtrate is stocked at 4°C [11], [19]. Under these conditions, they are stable for several years. Reactivity of each new batch is tested comparatively with the previous one using patients' sera with high, intermediate and low titers, as well as with a control negative sera and sera from patients with other fungal infections. Briefly, for the DID, glass slides (25×75 mm) were covered with melted purified agar gel punched according to a pattern (a central well surrounded by six wells). The antigen solution was placed in the central well while the peripheral wells were filled with the patient's sera and, as a positive control, either a patient's serum with a known positive titer or rabbit hyperimmune serum. Slides were incubated in a moist chamber at room temperature (20–25°C) and washed with 5% sodium citrate followed by 0.9% saline. They were dried and stained with Coomassie Brilliant Blue R (Sigma, USA). The CIE is also based on the diffusion of proteins but an electric current is applied through a buffered diffusion medium to accelerate the migration of antibody and antigen, with formation of the precipitation lines after around one hour. For the CIE, the glass slides were covered with 1% buffered agarose gel (pH 8.2) and two parallel rows of wells were punched in the gel. The patient's serum samples and positive control were applied to the anodic side and the antigens to the cathodic side of the slides. All sera were diluted two-fold in 0.9% saline and were tested from the undiluted sample. After electrophoresis, the slides were washed in 0.9% saline, dried and stained with Coomassie Brilliant Blue R. The differences in the protocols used by each center are detailed in Table 1.

**Table 1:** Details of the protocols used in the serological assays for paracoccidioidomycosis from the 6 reference centers.

Laboratory	Type of reaction	Duration of reaction	Type of buffer	Time of washing in saline	*P. brasiliensis* isolate(s) used for Ag preparation	Type of Ag/time of growth in culture
**IMTSP**	CIE^1^	90 m	Veronal^3^	48 h	IMTSP113/B339/IMTSP135	Crude filtrate/10 days
**FMRPUSP**	CIE	60 m	TEB^4^	24 h	Pb18/B339/BAT/BOAS	Sonicated/15 days
**UNESP**	CIE	90 m	Veronal	12 h	B339	Crude filtrate/10 days
**LEPAC**	IDD^2^	24 h	Distilled water	24 h	B339	Crude filtrate/7 days
**IALSP**	IDD	48 h	Distilled water	24 h	B339	Crude filtrate/10 days
**UNIFESP**	IDD	24 h	Distilled water	24 h	B339	Crude filtrate/10 days

1 ) Counterimmunoelectrophoresis.

2 ) Double Immunodiffusion.

3 ) Veronal buffer;

4 ) Tris- Borate-EDTA buffer.

### Scores and definition of discordances among the reference centers

All centers provided, as requested, 30 sera of PCM patients from their repository, collected within the last five years. These sera were then assayed blindly with regard to their titers by the other 5 centers. For this, the centers were randomly assigned A to F and the sera were numbered 1 to 30 by three of the authors (GMBDN, CPT, GB) who did not participate in the serological assays. To allow comparison among the centers' results, titers were transformed in scores ranging from 0 (negative) to 3 (high titers) according to each center's criteria as described in Table 2. Scores of the sera provided for this study ranged from 1 to 3, with score 1 corresponding to healing titers, and scores 2 and 3 corresponding to active disease with, respectively, low and high titers.

**Table 2:** Transformation of serological titers of the patients' sera into scores according to the criteria of each reference center.

Scores				
	0	1	2	3
**Centers**	Negative	Healing titers (inative disease)	Low titers (active disease)	High titers (active disease)
**A, D, E**	_	1∶1 to 1∶2	1∶4 to 1∶16	≥1∶32
**B, F**	_	1∶1 to 1∶4	1∶8 to 1∶32	≥1∶64
**C**	_	1∶1 to1∶16	1∶32 to 1∶64	≥1∶128

Each center's set of sera was assayed by the other five centers. The results from the donor center, arbitrarily defined as the reference score for their own sera, were then compared with the results of the other five centers. Discordance was defined as a different score, which could be minor, i.e., without a putative clinical consequence for the patient, or major, when the discordance could potentially lead to conflicting decisions regarding the patient's treatment. Minor discordances were between (a) scores 0 and 1: in both cases, either a negative serological result, or a low (healing) titer, would suggest inactive disease and both, in association with clinical and other data, eventually indicate treatment cessation; or (b) scores 2 and 3, both of which are associated with active disease. Major discordances were between scores 2 and 0 or 1, and between 3 and 0 or 1, which, when associated with proper clinical and other laboratorial data, may have led to a different treatment of the patient. Comparisons among laboratories were done using the Chi-square and Fischer exact test. Differences were considered significant when *p*<0.05.

## Results and Discussion

All centers exhibited a surprisingly high rate of “major” discordances when the scores given by each center to the sera provided by the other 5 centers were compared with the “reference” scores (Table 3). There was some variability in the rate of discordances among the centers, ranging from 22 (15%) to 45 (30%) “major” discordant scores out of 150 scores given, and a mean number of discordant scores of 31 (20%). In fact, the rates of discordances differed significantly among the centers (*p* = .0007, Chi-square). Minor discordances were also highly frequent, ranging from 16 to 52 out of 150 scores given and a mean of 36 (24%) (Table S1).

**Table 3:** Comparison of the scores from the donor center (reference score) given to their own sera with the scores given by the other five centers.

*n* of scores with major discordance with the score provided by each reference center						
Centers	A	B	C	D	E	F
**A** (*n* = 30)	-	5	8	9	6	2
**B** (*n* = 30)	11	-	5	7	4	5
**C** (*n* = 30)	8	3	-	14	7	7
**D** (*n* = 30)	13	7	9	-	9	6
**E** (*n* = 30)	5	5	3	8	-	2
**F** (*n* = 30)	3	3	2	7	8	-
**Total**	40/150	23/150	24/150	45/150	34/150	22/150

Analysis of the performance using the scores given by one center to their own sera (reference score) and comparing them to the scores given to their sera by the remaining five other centers, showed a high rate of major discordances as well (Table 4). For example, 15 out of the 30 (50%) center A's reference scores were discordant with at least one of the remaining centers' scores, and, eight out of the 30 reference scores (27%) were discordant with two or more of the remaining centers. Again variability in the rates of discordance was detected among the centers: in the first comparison, it ranged from 9 (30%) to 23 (77%) scores (*p* = .0157, chi-square) and for the second comparison it ranged from 2 (7%) to 10 (33%) scores (*p*>0.05, Chi-square). The mean numbers of sera presenting a “major” discordance were respectively 14.8 and 7.1. In all, considering the 180 references scores provided by the 6 centers to their own sera, 79 (44%) of them presented a major discordance with at least one of the other center's score, and 43 (24%) presented a major discordance with at least two other centers' scores (Table 4). Minor discordances were also frequent when this other analysis was used: for the reference center A, “minor” discordances with at least one other center were found for 19 of their sera (Table S2). In all, 95 of the 180 sera (53%) presented a “minor” discordance with at least one other laboratory result (Table S2).

**Table 4:** *n* of major discordant scores with at least one or two other centers.

Centers providers of the sera with reference scores	*n* of major discordant scores with	
	at least one center	at least two centers
**A**	15/30	8/30
**B**	14/30	9/30
**C**	15/30	9/30
**D**	23/30	10/30
**E**	9/30	2/30
**F**	13/30	5/30
**Total**	89/180	43/180

The 6 control negative sera provided by one of the centers were also negative (score 0) when assayed by the other 5 centers, with the exception of one serum that received a score 1 (titer 1∶2) by laboratory C. This titer is consistent with a healing titer or a non-specific reaction according to this laboratory criterion.

Since each lab has its own, in house, assay for detection of anti-*P. brasiliensis* antibodies, we anticipated that “minor” discordances (i.e., slight and clinically not relevant differences in the titers of antibodies) would occur with some frequency. Unexpectedly, we found a high rate of “major” discordances (i.e. differences in scores that may have led to different clinical managements: maintenance or interruption of the treatment). In an attempt to understand the reasons for these discrepancies, the influence of two main variables that discriminated the centers with regard to their protocols were evaluated, namely the technique employed (DID [n = 3 centers] vs. CIE [n = 3]) and type of the antigen (single *P. brasiliensis* isolate [n = 4 centers] vs. pool of isolates [n = 2]). Gathering the 150 scores given by each one of the 3 centers performing the DID technique to the 5 other centers' sera, of a total of 450 scores, in 343 instances there was agreement and in 107 major discordance; the same analysis for the 3 centers using the CIE technique showed more concordant scores (n = 369) and less discordant scores (n = 81, *p* = 0.04, Fischer exact test). Among the 300 scores given by the 2 centers using a pool of isolates, the proportion was 47 discordant and 253 concordant scores. This proportion was significantly higher than that obtained with the 4 centers using only one isolate: 141 discordant and 459 concordant scores (*p* = 0.007, Fischer exact test). Thus, the type of the reaction and antigen preparation may be factors that influence the accuracy of the serological result. Regarding the antigen preparation, not only gp43, but several other components in both the somatic and culture filtrate antigens react with the patients' sera [20], [21], [22]. The amount of these components in the antigen preparations not only varies among the strains, but also in a single strain depending on the number of repeated subculturing, medium used, log phase of growth when the fungus is harvested, among other factors. This is probably a major factor in the inconsistencies among centers. Other particularities that likely influenced the accuracy of the serological results (such as duration of reaction, incubation time, expertise and background of the technician responsible for performing the assay, etc.), could not be assessed in the present study because it was not designed to evaluate these factors.

The present study demonstrated a high rate of discordance among centers that are considered to be reference centers for the diagnosis and serological follow up of PCM patients. Due to the fact that, per request, only sera from PCM patients were provided by these centers, we could not analyze the performance of the serological tests for the diagnosis of PCM. For this, sera of patients with other mycoses and infectious diseases would also be required. However, the high rate of discordances found certainly raises some suspicion with regard to this issue. We illustrate this possibility with one of the sera from center B, whose donor was a 56 year-old patient with chronic non-specific respiratory symptoms, initially and presumptively diagnosed as pulmonary tuberculosis (TB) at a community health care unit. TB treatment was ineffective, the pulmonary symptoms worsened and he developed a pneumothorax. Microbiological evaluation was negative on both sputum and bronchoalveolar lavage. Diagnosis of PCM was made based on the history of having lived in an endemic area, a suggestive chest X-ray, and a 1∶32 titer on the CIE test for PCM (score 2, active disease). A similar active disease score was given by centers E and F, but centers A, C and D gave titers corresponding to score 1 (1∶1, 1∶8 and 1∶2, respectively), suggestive of healing disease, which could potentially delay the diagnosis and the beginning of antifungal treatment.

Relapses and recrudescence are commonly reported during the prolonged (usually >1 year) antifungal therapy of this mycosis. In Argentina, Negroni et al [23] reported that 14.3% of the patients relapsed after a follow up of 15 months. In Brazil, Marques reported 13.8% of relapses after 10 years of follow up, although almost half of the relapses occurred in the first 3 years, when the patients were still on or just off antifungal therapy [24]. Serological follow up has been shown to be an important tool in the early diagnosis of relapses [13], [14], [25]. The factor most commonly reported as contributing to the failure of the anti-fungal treatment is poor compliance due to socio-economical factors such as alcoholism, unemployment and/or long distance from the local drug provider [26]. Although decisions regarding the interruption or prolongation of drug therapy are not made solely based on the serological result, we speculate that in certain cases the relapses would be related to inadvertent therapy discontinuation due to misleading serological monitoring. On the other hand, some patients may undergo unnecessary prolongation of the antifungal therapy. In any case, it is clear from the present study that an effort from the medical mycology community must be undertaken (or re-undertaken) to improve better standardization of the serological diagnosis of this mycosis. Our results suggest that particularly the type of antigen (pool vs. single isolate) and technique (DID vs. CIE) should be addressed.

Efforts should also be made at the same time to develop and standardize *P. lutzii* serological diagnosis tests. This is a new species in the *Paracoccidioides* genera recently described that is endemic in some areas of South America where the patients' sera were reported to not recognize the *P. brasiliensis'* antigens in conventional serological tests [27]–[29]. This issue could not be addressed here since the 6 reference centers participating in the study were located in *P. brasiliensis* endemic areas and provided sera only with positive serological results. However, occasionally reference centers outside *P. lutzii* endemic areas may handle sera from PCM due to *P. lutzii* and release false negative serological results. This has already been documented [30] and will certainly be more common owing to the increasing migration rates in South American countries, particularly Brazil.

Finally, high discordance rates may well occur in the diagnosis of other endemic mycoses such as histoplasmosis, coccidioidomycosis and blastomycosis, all of which are endemic in some areas of South America and that are covered by the reference centers involved in this study or by other reference centers. The efforts to improve the serological diagnosis should also be addressed for these mycoses that, like PCM, remain among the most neglected diseases in South America.

## Supporting Information

Checklist S1 STARD checklist.
(DOC)

Table S1 Number of scores with minor discordance with the score provided by each reference center.
(DOCX)

Table S2 Number of scores with minor discordance with at least one other center.
(DOCX)
