# Supplementary material for: Serological Diagnosis of Paracoccidioidomycosis: High Rate of Inter-laboratorial Variability among Medical Mycology Reference Centers
Source: PLoS Negl Trop Dis. 2014 Sep 11;8(9):e3174. doi: 10.1371/journal.pntd.0003174 (PMC4161321; doi:10.1371/journal.pntd.0003174)
Supplement: Table S1 — Number of scores with minor discordance with the score provided by each reference center. (DOCX) [file pntd.0003174.s002.docx]

**Supplementary table S1:** *n* of scores with minor discordance with

the score provided by each reference center

| major discordance | | | | | | |
| --- | --- | --- | --- | --- | --- | --- |
| Sera from reference center | **A** | **B** | **C** | **D** | **E** | **F** |
| A | _ | 4 | 6 | 10 | 14 | 7 |
| B | 15 | _ | 5 | 13 | 13 | 6 |
| C | 11 | 3 | _ | 9 | 6 | 7 |
| D | 3 | 4 | 8 | _ | 6 | 3 |
| E | 10 | 4 | 5 | 12 | _ | 6 |
| F | 10 | 1 | 3 | 8 | 4 | _ |
| Total | 49/150 | 16/150 | 27/150 | 52/150 | 43/150 | 29/150 |
