# Supplementary material for: Serological Diagnosis of Paracoccidioidomycosis: High Rate of Inter-laboratorial Variability among Medical Mycology Reference Centers
Source: PLoS Negl Trop Dis. 2014 Sep 11;8(9):e3174. doi: 10.1371/journal.pntd.0003174 (PMC4161321; doi:10.1371/journal.pntd.0003174)
Supplement: Table S2 — Number of scores with minor discordance with at least one other center. (DOCX) [file pntd.0003174.s003.docx]

**Supplementary table S2:** *n* of minor discordant scores

with at least one other center

| Reference center | minor discordant |
| --- | --- |
| A | 19/30 |
| B | 16/30 |
| C | 14/30 |
| D | 13/30 |
| E | 17/30 |
| F | 16/30 |
| Total | 95/150 |
